# Synergistic effects of early sequential enhanced external counterpulsation and electric tilt table training on functional recovery in ischemic stroke: study protocol of a randomized controlled trial

**DOI:** 10.3389/fneur.2026.1842199

**Published:** 2026-07-14

**Authors:** Liu Shi, Jiagui Ma, Xilian Hu, Zhaohui Jin, Zhenying Zhang, Zishuang Liu, Yanyan Yin, Xiaojing Sun, Cheng Yang, Weitao Wang, Yanjun Liu, Tiejun Liu

**Affiliations:** Beijing Rehabilitation Hospital, Capital Medical University, Beijing, China

**Keywords:** electric tilt table, enhanced external counterpulsation, ischemic stroke, neurorehabilitation, study protocol

## Abstract

**Background:**

Ischemic stroke remains a leading cause of disability worldwide. Early rehabilitation is crucial for functional recovery. Electric tilt table (ETT) training improves motor and balance function and facilitates early mobilization in bedridden patients, but its use is often limited by orthostatic hypotension (OH). Enhanced external counterpulsation (EECP), a noninvasive circulatory support technique, has shown benefits in cardiovascular and cerebrovascular diseases by improving vascular function and cardiac output. Given its hemodynamic effects, EECP may enhance tolerance to verticalization. However, whether early sequential application of EECP followed by ETT training improves functional recovery after ischemic stroke remains unclear. This study aims to determine whether this sequential strategy improves neurological and functional recovery while reducing symptomatic orthostatic hypotension in patients with ischemic stroke.

**Methods:**

This single-center, randomized controlled clinical trial will be conducted at Beijing Rehabilitation Hospital, Capital Medical University. A total of 104 participants with ischemic stroke within 3 months of onset will be enrolled and randomly allocated in a 1:1 ratio to either the ETT training group (conventional rehabilitation plus once-daily 30-min ETT training) or the sequential EECP - ETT training group (conventional rehabilitation plus once-daily 60-min EECP immediately followed by 30-min ETT training). Both interventions will be delivered 5 days per week for 7 weeks. The primary outcome is overall neurological function at week 7, assessed by the National Institutes of Health Stroke Scale (NIHSS). Secondary outcomes include the incidence of symptomatic OH during ETT training sessions, motor function (Fugl-Meyer Assessment, FMA), balance (Berg Balance Scale, BBS), swallowing (Modified Mann Assessment of Swallowing Ability, MMASA), activities of daily living (Barthel Index, BI), and noninvasive hemodynamic parameters (cardiac index, stroke volume, systemic vascular resistance index) assessed at week 7. Outcome assessments will be performed by blinded evaluators. Safety will be monitored throughout the study period.

**Discussion:**

This study will evaluate the sequential application of EECP and ETT in early ischemic stroke rehabilitation. The findings will clarify whether this strategy safely alleviates symptomatic orthostatic hypotension and promotes overall functional recovery, potentially offering an effective and integrative approach for early stroke intervention.

**Trial registration:**

http://www.chictr.org.cn, Chinese Clinical Trial Registry, ChiCTR2400083761. Registered on April 30, 2024.

## Background

Ischemic stroke remains a major public health challenge worldwide, characterized by high incidence, substantial disability, and considerable mortality (1, 2). While early systematic rehabilitation is essential for functional recovery, traditional motor therapies offer limited efficacy (3, 4). For bedridden patients, early verticalization is imperative to restore consciousness, activate musculature, and improve motor stability (5–7). Although the electric tilt table (ETT) training is a standard tool for verticalization, its utility is often constrained by orthostatic hypotension (OH) (8–11). OH is common during inpatient stroke rehabilitation, affecting nearly half of patients; symptomatic OH occurs in approximately one-third of patients (12). Because ETT training involves progressive verticalization, OH may be precipitated or exacerbated during training. Symptomatic OH is of particular concern because it may interrupt rehabilitation sessions, limit tolerance to early mobilization, and compromise rehabilitation progress (13). Given the lack of definitive treatments for OH, targeted pre-conditioning strategies are required to enhance orthostatic tolerance (14).

Enhanced external counterpulsation (EECP) is a noninvasive circulatory support technique that augments venous return and cardiac output, improving perfusion to the heart and brain (15–18). Beyond its established therapeutic benefits, EECP’s hemodynamic effects suggest potential as a pre-conditioning strategy. By stabilizing vascular tone and increasing systemic perfusion prior to verticalization, EECP may mitigate OH and improve tolerance to ETT training (19). However, evidence regarding the sequential integration of these modalities remains scarce.

**Figure 1:**
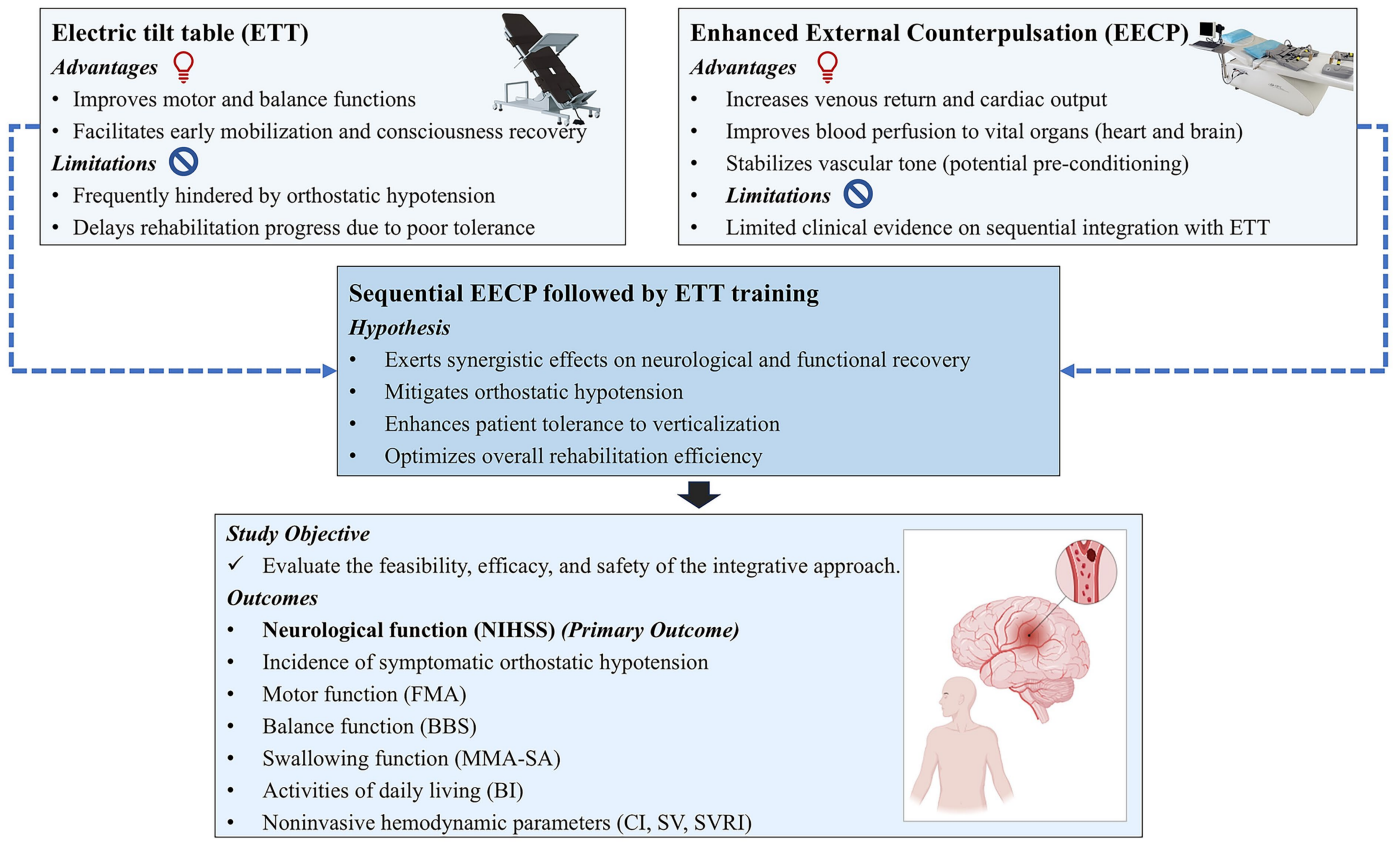
Conceptual framework of the study design. BBS, Berg Balance Scale; BI, Barthel Index; CI, Cardiac Index; EECP, Enhanced External Counterpulsation; ETT, Electric tilt table; FMA, Fugl-Meyer Assessment; MMASA, Modified Mann Assessment of Swallowing Ability; NIHSS, National Institutes of Health Stroke Scale; SV, Stroke Volume; SVRI, Systemic Vascular Resistance Index.

Consequently, we hypothesize that the early sequential application of EECP followed by ETT training synergistically promotes neurological and functional recovery in patients with ischemic stroke. This study aims to evaluate the clinical feasibility, efficacy, and safety of this integrative approach. We will assess its impact on neurological, motor, balance, and swallowing functions, alongside activities of daily living and noninvasive hemodynamic parameters (Figure 1). If validated, this strategy could optimize rehabilitation efficiency, providing evidence for a safe and accelerated early recovery pathway in stroke patients.

## Methods

### Study design

**Figure 2:**
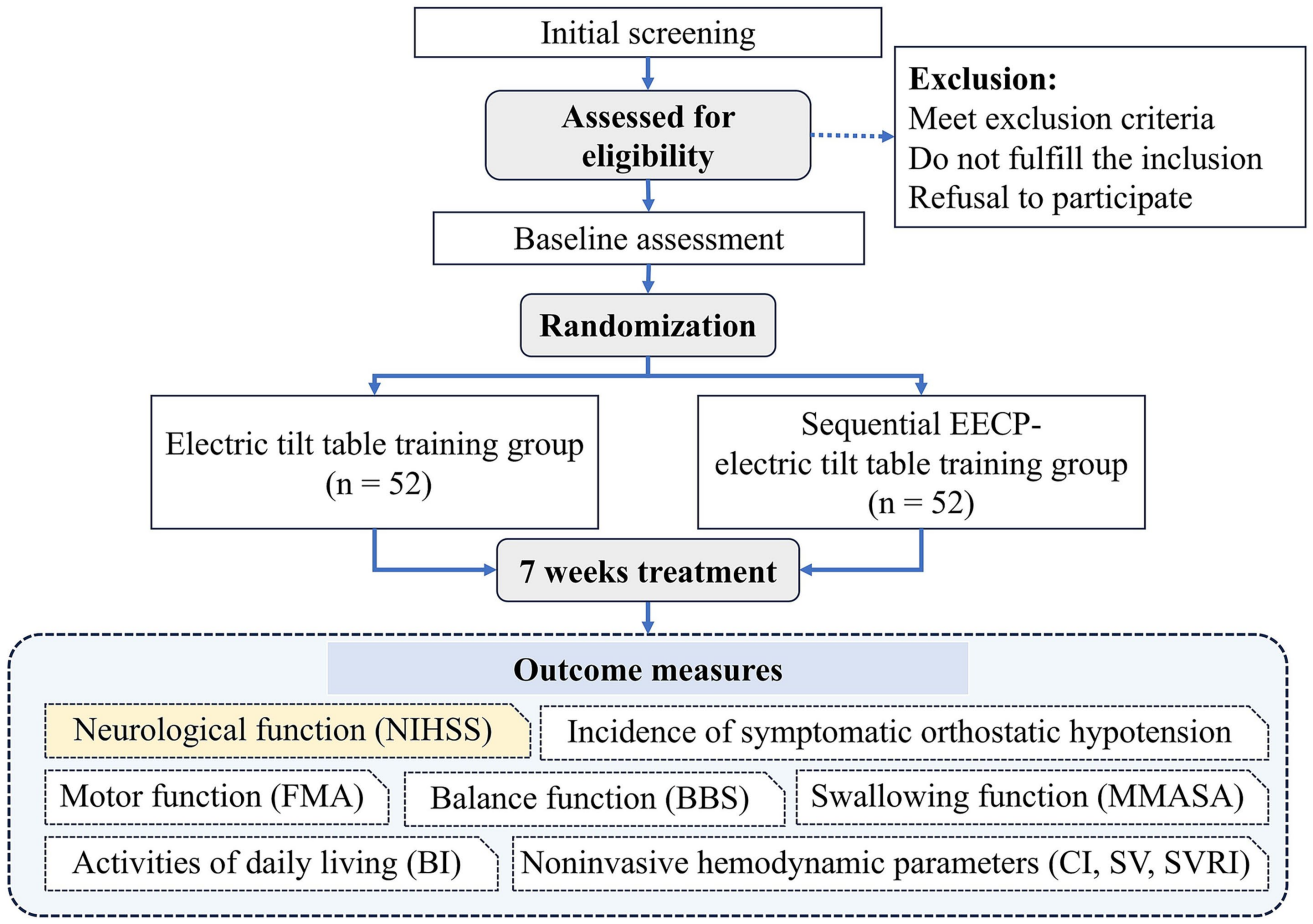
Study flowchart of trial process. BBS, Berg Balance Scale; BI, Barthel Index; CI, Cardiac Index; EECP, Enhanced External Counterpulsation; FMA, Fugl-Meyer Assessment; MMASA, Modified Mann Assessment of Swallowing Ability; NIHSS, National Institutes of Health Stroke Scale; SV, Stroke Volume; SVRI, Systemic Vascular Resistance Index.

This single-center, randomized controlled clinical trial will be conducted at Beijing Rehabilitation Hospital, Capital Medical University, and has been registered with the Chinese Clinical Trial Registry (ChiCTR2400083761). The protocol adheres to the Standard Protocol Items: Recommendations for Interventional Trials guidelines (20), the Declaration of Helsinki (21). It has been approved by the institutional Ethics Committee and will be overseen by the Department of Scientific Research Management of the Beijing Rehabilitation Hospital, Capital Medical University. Following the acquisition of written informed consent, enrolled participants will undergo a 7-week study period. The detailed trial workflow is depicted in Figure 2.

### Participants

Patients diagnosed with ischemic stroke undergoing rehabilitation at Beijing Rehabilitation Hospital, Capital Medical University, will be screened for eligibility.

Eligible participants must meet all of the following inclusion criteria: (1) aged ≥ 18 years; (2) first-ever stroke, or recurrent stroke without residual limb motor or consciousness deficits attributable to the previous stroke, with onset time <3 months; and (3) provision of written informed consent by the patient or a legally authorized representative.

Candidates will be excluded from the study if they meet any of the following criteria: (1) hemorrhagic disorders or the use of oral anticoagulants posing a bleeding risk; (2) rheumatic heart disease; (3) moderate-to-severe aortic regurgitation and/or stenosis; (4) aortic dissection, thoracoabdominal/cerebral aneurysm, or vascular malformation; (5) moderate-to-severe pulmonary hypertension, right ventricular dysfunction, or decompensated left heart failure; (6) uncontrolled severe hypertension (blood pressure >170/110 mmHg); (7) uncontrolled arrhythmias, (e.g., frequent premature contractions, ectopic tachycardia, second- or third-degree atrioventricular block, or other clinically significant rhythm disturbances); (8) active thrombophlebitis or deep vein thrombosis; (9) local skin infection or moderate-to-severe edema at the cuff placement sites; (10) severe symptomatic peripheral vascular disease or prior lower-limb arterial stenting implantation; (11) active malignancy; or (12) pregnancy.

Eligibility will be determined by the investigators based on participants’ medical history, review of medical records, routine laboratory tests, and pre-enrolment clinical assessments. All participants will undergo electrocardiography, 24-h Holter monitoring, transthoracic echocardiography, and lower-extremity vascular ultrasonography before enrolment. Additional examinations will be performed when clinically indicated to confirm eligibility or exclude potential contraindications.

Participants will be withdrawn from the trial under any of the following conditions: (1) withdrawal of informed consent; (2) requirement for prohibited concomitant therapies or procedures that may confound study outcomes; or (3) loss to follow-up or relocation precluding scheduled assessments.

### Randomization and blinding

Enrolled participants will be randomly assigned in a 1:1 ratio to either the ETT training group (receiving conventional rehabilitation combined with ETT training) or the sequential EECP-ETT training group (receiving conventional rehabilitation combined with sequential EECP followed by ETT training). The randomization sequence will be computer-generated by an independent statistician using SPSS software, employing a variable block randomization design. Randomization will not be stratified according to any baseline characteristics. To ensure strict allocation concealment, sequentially numbered, opaque, sealed envelopes will be utilized. Due to the physical nature of the interventions, blinding of participants and administering therapists is unfeasible. However, to minimize detection bias, all outcome assessors and data analysts will remain strictly blinded to the group assignments throughout the trial.

### Clinical data collection

Comprehensive baseline data will be collected for all enrolled participants, encompassing the following categories:

Demographic characteristics: age, sex, height, and weight (with body mass index [BMI] calculated accordingly).

Stroke-related specifications: date of ischemic stroke onset, infarct location, history of reperfusion therapy (e.g., intravenous thrombolysis or mechanical thrombectomy), and related surgical interventions (e.g., decompressive craniectomy).

Comorbidities: presence of hypertension, diabetes mellitus, myocardial infarction, cardiomyopathy, and specific arrhythmias.

Current medications: use of antiplatelet agents, anticoagulants, statins, antihypertensive, and neurotrophic/neuroprotective agents.

Vital signs: body temperature, blood pressure, heart rate and rhythm, respiratory rate, and peripheral oxygen saturation.

Laboratory parameters: white blood cell count, neutrophil percentage, hemoglobin, platelet count, interleukin-6, C-reactive protein, procalcitonin, D-dimer, B-type brain natriuretic peptide, alanine aminotransferase, aspartate aminotransferase, serum creatinine, blood urea nitrogen, albumin, and prealbumin.

### Rehabilitation assessments

Rehabilitation assessments will be performed across multiple domains, including consciousness, cognitive and language function, motor and sensory function, swallowing ability, hemodynamic status, bladder and bowel function, activities of daily living, and psychological status. The following standardized instruments will be specifically utilized: the National Institutes of Health Stroke Scale (NIHSS) for neurological impairment (22, 23), the Fugl-Meyer Assessment (FMA) for motor function (24), the Berg Balance Scale (BBS) for balance function (25), the Modified Mann Assessment of Swallowing Ability (MMASA) for deglutition (26), and the Barthel Index (BI) for activities of daily living (27). Additionally, noninvasive hemodynamic parameters will be evaluated via an assessment system based on thoracic electrical bioimpedance (28, 29).

### Interventions

All participants will receive standard of care, consisting of conventional rehabilitation therapy and comprehensive nursing care. Specific interventions related to the study groups will be administered in addition to this baseline therapy.

Monitoring and Nursing Care: Participants will undergo continuous clinical observation and hemodynamic monitoring. Standardized nursing protocols will be implemented, including the management of indwelling catheters, anti-spasticity limb positioning, nutritional support, medication reconciliation, and psychological counseling.

Rehabilitation therapy: Personalized rehabilitation programs will be formulated by a multidisciplinary team, incorporating data from physical examinations, laboratory results, and functional assessments. Depending on clinical indications, therapeutic modalities may include acupuncture, Chinese therapeutic massage (Tuina), swallowing therapy, cognitive rehabilitation, and speech-language interventions. Neurological status and functional progress will be systematically reassessed to titrate treatment intensity and adjust rehabilitation plans accordingly.

Communication and education: To ensure treatment adherence, clinicians will provide structured health education to patients and their caregivers, covering disease progression and safety precautions. Formal informed consent will be obtained prior to the initiation of each specific therapeutic modality.

1. ETT training group

Prior to the intervention, participants will be briefed on the principles and safety precautions of the ETT training to alleviate anxiety and optimize compliance. Patients will be positioned supine on the tilt table, with the thorax, pelvis, and knees secured by safety straps. A support board will be utilized for upper limbs stabilization, while the lower limbs remain extended with the feet resting firmly against the footboard. Participants will be instructed to maintain a relaxed, neutral posture throughout the procedure.

The training will initiate at a tilt angle of 30°. Following an initial 15-min tolerance period, the angle will be increased in increments of 10–15°, as tolerated by the patient. Each session will last 30 min, performed once daily, 5 days per week. Both the tilt angle and duration will be progressively adjusted based on the patient’s functional recovery. The primary therapeutic goal is to achieve tolerance of a 90° vertical position for 30 min without adverse symptoms or discomfort.

For safety monitoring, hemodynamic and respiratory parameters, including systolic blood pressure, diastolic blood pressure, mean arterial pressure, heart rate, and oxygen saturation, will be recorded prior to each session, monitored continuously during training, and recorded again immediately upon completion of the session.

1. Sequential EECP - ETT training group

Participants in this group will receive EECP therapy followed by ETT training in a sequential manner.

EECP therapy will be initiated following standard equipment calibration and safety checks (including cuff integrity and system initialization). Electrodes will be positioned at designated precordial sites to ensure stable ECG triggering, and a digital plethysmographic pulse oximeter will be attached for real-time hemodynamic monitoring. Participants will be positioned supine, with pressure cuffs wrapped sequentially around the calves, lower thighs, and upper thighs/buttocks, ensuring the coccyx is correctly aligned.

EECP inflation pressure will start at the minimum effective level and be incrementally uptitrated to the target therapeutic range over 3–5 sessions, adjusted for individual subcutaneous fat and muscle mass. In this study, the target inflation pressure is 150 mmHg. Treatment efficacy will be optimized by adjusting the inflation/deflation pressure and timing to maintain a diastolic-to-systolic augmentation ratio (D/S ratio) > 1.2 and a diastolic-to-systolic area ratio (DP/SP ratio) of 1.5–2.0. Each session will last 60 min, though duration may be reduced based on patient tolerance. Training will be conducted 5 h per week for 7 weeks, totaling 35 sessions.

Electric tilt table training will be initiated exactly 15 min after the completion of each EECP session. Systolic and diastolic blood pressure, mean arterial pressure, heart rate, and oxygen saturation will be recorded before, during, and after both EECP and ETT training.

Safety considerations: Potential transient adverse effects associated with EECP and ETT training include lower-limb discomfort, skin erythema or abrasion at the cuff contact sites, transient headache, dizziness, nausea, excessive sweating, orthostatic intolerance, and syncope. All adverse events will be documented throughout the study and managed according to predefined safety procedures. Participants who develop serious or intolerable intervention-related adverse events (e.g., refractory orthostatic hypotension, persistent arrhythmias, decompensated heart failure, or syncope requiring medical evaluation) or new contraindications to EECP or ETT (e.g., incident venous thrombosis or progression of aortic regurgitation), as determined by the investigators, will discontinue the study intervention. Unless they withdraw informed consent, these participants will continue scheduled follow-up assessments according to the study protocol. Training (either EECP or ETT) will be immediately discontinued if any of the following clinical conditions occur: marked fatigue, acute pain, agitation, profuse sweating, palpitations, chest tightness or angina, dyspnea, heart rate ≥120 bpm or ≤40 bpm, systolic blood pressure >180 mmHg or <90 mmHg, respiratory rate ≥35 breaths/min, oxygen saturation <90%, new-onset arrhythmias, or dynamic ST–T segment changes. Standardized medical management will be promptly initiated.

### Assessment schedule and retention strategies

Baseline assessments will be performed upon enrollment. Post-intervention assessments will be conducted immediately following the completion of the 7-week treatment period. All scheduled evaluations will be executed according to the predefined study timeline and standardized protocols. To enhance participant adherence and ensure data integrity, the research team will implement a proactive engagement strategy. This includes longitudinal monitoring through bedside visits (for inpatients) and regular telephonic follow-ups (for discharged patients or caregivers). These interactions are designed to monitor recovery progress, mitigate potential attrition (dropout), and promptly address any concerns or queries regarding the study procedures. Measures to minimize missing data will include double-checking assessment records and maintaining flexible scheduling for follow-up evaluations.

### Outcomes

The outcome measures were selected to evaluate the potential clinical benefits of the sequential EECP-ETT strategy within a comprehensive rehabilitation program. As the proposed sequential intervention is intended to improve orthostatic tolerance and facilitate early verticalization, its potential benefits are expected to extend beyond immediate hemodynamic effects to multiple domains of functional recovery commonly affected after stroke. The primary outcome is neurological function at week 7, assessed by the NIHSS. NIHSS was selected as the primary endpoint because it comprehensively reflects overall neurological recovery, which is the ultimate goal of early stroke rehabilitation beyond immediate hemodynamic improvements. Secondary outcomes include the incidence of symptomatic OH, recorded continuously during all ETT training sessions throughout the 7-week intervention. OH is defined as a systolic blood pressure drop ≥ 20 mmHg, a diastolic drop ≥ 10 mmHg, or the onset of hypotensive symptoms within 3 min of tilting (30). Additional secondary outcomes, assessed at week 7 (Table 1), evaluate specific functional domains and hemodynamics, including motor function quantified by the FMA, balance function assessed by the BBS, swallowing ability via the MMASA, activities of daily living quantified by the BI, and noninvasive hemodynamic parameters, specifically cardiac index (CI), stroke volume (SV), and systemic vascular resistance index (SVRI).

**Table 1:** Outcome domains and measurement instruments.

**Outcome domain**	**Measurement instrument**	**T0**	**T1**
Primary outcome			
Neurological function	National Institutes of Health Stroke Scale	√	√
Secondary outcomes			
incidence of symptomatic orthostatic hypotension	Noninvasive blood pressure monitor and clinical observation	*	*
Motor function	Fugl-Meyer Assessment	√	√
Balance function	Berg Balance Scale	√	√
Swallowing function	Modified Mann Assessment of Swallowing Ability	√	√
Activities of daily living	Barthel Index	√	√
Noninvasive hemodynamic parameters	Thoracic electrical bioimpedance assessment system	√	√

T0 baseline, T1 post-intervention (week 7). *The incidence of symptomatic orthostatic hypotension is monitored continuously during each ETT training session rather than at discrete T0/T1 timepoints.

### Participant timeline

Timeline for participants’ enrolment, interventions, and assessments is shown in Table 2.

**Table 2:** Time schedule of enrolment, interventions and assessments.

	**Study period**								
	**Enrolment**	**Allocation**	**Post-allocation**						
**Timepoint**	**-t1**	**t0**	**t1**	**t2**	**t3**	**t4**	**t5**	**t6**	**t7**
Enrolment									
Eligibility screen	X								
Informed consent	X								
Allocation		X							
Pre-treatment preparation									
Rehabilitation assessments			X						
Intervention									
A: Electric tilt table training			X	X	X	X	X	X	X
B: Sequential EECP-electric tilt table training group			X	X	X	X	X	X	X
Assessments									
National Institutes of Health Stroke Scale			X						X
Symptomatic orthostatic hypotension			X	X	X	X	X	X	X
Fugl-Meyer Assessment			X						X
Berg Balance Scale			X						X
Modified Mann Assessment of Swallowing Ability			X						X
Barthel Index			X						X
Noninvasive hemodynamic parameters			X						X
Safety			X	X	X	X	X	X	X

t1–t7 correspond to intervention weeks 1–7; EECP, Enhanced External Counterpulsation.

### Sample size estimation

Since there is limited literature evaluating the synergistic effects of this specific sequential intervention, the sample size was calculated based on our unpublished preliminary pilot study involving 18 participants (9 in each group). The mean NIHSS score at the end of the trial was 7.99 ± 3.82 in the ETT training group and 5.28 ± 2.16 in the sequential EECP-ETT training group, giving an expected difference (*δ*) of 2.71 with a standard deviation (*σ*) of 3.82. With a two-sided significance level (*α*) of 0.05, power (1–*β*) of 90%, and equal allocation (1: 1), the required sample size for each group was calculated using the formula:


$$n=\frac{2{\left(z_{\alpha }+z_{\beta }\right)}^{2}*\sigma^{2}}{\delta^{2}}$$

This calculation indicated that 42 participants per group were required (84 in total). Allowing for a 20% dropout rate, 52 participants will be recruited per group, for a total sample size of 104.

### Statistical analysis

Continuous variables will be summarized as mean ± standard deviation if normally distributed or as median (interquartile range) otherwise. Categorical variables will be presented as frequencies and percentages. Group comparisons will be performed using the *t*-test for normally distributed continuous variables, the Wilcoxon rank-sum test for non-normally distributed data, and the chi-square or Fisher’s exact test for categorical variables. To account for potential baseline imbalances, group differences in primary and secondary outcomes at week 7 will be evaluated using Analysis of Covariance (ANCOVA), with the respective baseline scores included as covariates.

Analyses will be performed on both the intention-to-treat (ITT) and per-protocol (PP) populations, with the ITT set serving as the primary analysis population. Missing data in the ITT population will be handled using multiple imputation. Safety will be assessed in all participants receiving at least one intervention session. Adverse events and complications will be analyzed using chi-square or Fisher’s exact tests.

Exploratory subgroup analyses according to baseline stroke severity (baseline NIHSS score) will be performed to explore potential heterogeneity of treatment effects. Because the study is not powered for subgroup analyses, these analyses will be considered exploratory and interpreted cautiously.

All statistical analyses will be conducted using R software (version 4.2.2; R Foundation for Statistical Computing, Vienna, Austria). All tests will be two-sided with a significance level of 0.05. Statisticians will remain blinded to group allocation throughout the process.

### Data collection and management

Baseline demographic and clinical characteristics will be recorded solely at enrollment, whereas functional assessments will be evaluated at both baseline and the end of the 7-week intervention period. Hemodynamic parameters (blood pressure, heart rate, mean arterial pressure, and oxygen saturation) will be monitored and documented before, during, and after each intervention session. All clinical data will be entered into standardized electronic Case Report Forms (eCRFs) by trained personnel. To ensure data integrity, a secure Electronic Data Capture (EDC) system will be utilized. Data quality will be guaranteed through real-time automated logic and range checks, supplemented by a double-data entry protocol and routine cross-validations by an independent data manager. To strictly protect patient confidentiality, all participants will be de-identified using unique study-assigned codes. The EDC system incorporates a comprehensive audit trial, logging all modifications with timestamps and operator identifications to ensure complete traceability. All documentation and data storage will rigorously comply with institutional and national data protection regulations.

## Discussion

Early mobilization via ETT-mediated verticalization is an integral component of stroke rehabilitation, particularly for patients with severe motor deficits (31, 32). Transcending passive positioning, verticalization serves as a potent physiological stimulus that activates the reticular activating system to enhance arousal, heighten proprioceptive input, and facilitate orthostatic adaptation (33–35). Additionally, ETT training is a key clinical strategy to mitigate the sequelae of prolonged bed rest, such as cardiopulmonary deconditioning and venous stasis (36). However, its clinical utility is frequently constrained by OH. This failure of autonomic adjustment to gravitational stress often leads to training interruptions, thereby limiting mobilization intensity and potentially delaying functional recovery (37). Addressing this orthostatic barrier is therefore a clinical priority for optimizing rehabilitation outcomes.

Enhanced external counterpulsation offers a physiological solution to this dilemma. Building upon its established utility in cardiology, EECP theoretically establishes a ‘hemodynamic priming’ state in stroke patients through its mechanisms of diastolic augmentation and enhanced venous return (38–40). By increasing shear stress and improving endothelial function, EECP actively redistributes pooled blood from the splanchnic and lower-limb prior to gravitational challenge (41, 42). We hypothesize that this pre-conditioning stabilizes systemic vascular resistance and maintains sustained cerebral perfusion pressure during subsequent ETT training. EECP may enhance verticalization tolerance in terms of both duration and angle, thereby ensuring patients receive the optimal therapeutic intensity required for early recovery.

This trial is designed to provide preliminary clinical evidence regarding the sequential integration of these two modalities within a sequential framework. Unlike isolated therapies, the “EECP followed by ETT” strategy targets a synergistic effect: EECP optimizes the hemodynamic substrate, while ETT training capitalizes on this stability to maximize motor and neurological relearning. If this strategy successfully improves orthostatic tolerance and enables patients to tolerate earlier and more intensive verticalization training, benefits may extend beyond overall neurological recovery to multiple functional domains. Early verticalization may provide weight-bearing, proprioceptive input, and postural activation of the affected limb, thereby facilitating motor recovery and balance, which will be evaluated using the FMA and BBS. In addition, improved overall neurological recovery and earlier comprehensive rehabilitation may also contribute to better swallowing function and greater independence in activities of daily living, as assessed by the MMASA and BI. Because orthostatic intolerance and impaired mobilization may occur across a broad spectrum of patients during early stroke rehabilitation, the present trial does not restrict enrolment according to baseline stroke severity. Although patients with greater mobility impairment may derive greater benefit from improved orthostatic tolerance during verticalization training, patients with milder stroke may also benefit through enhanced participation in early rehabilitation. This study will not only verify the safety and feasibility of this integrative approach but also elucidate whether stabilizing hemodynamics correlates with superior functional outcomes. If successful, this sequential protocol could potentially refine the standard of care by defining a safe, accelerated pathway for early mobilization, thereby improving the management of bedridden ischemic stroke patients.

Several limitations to this study should be acknowledged. First, as a single-center randomized controlled trial, the generalizability of our findings to diverse clinical settings or more severe stroke populations may be limited. Future multi-center studies with larger, more heterogeneous cohorts are warranted to validate the efficacy of this sequential protocol. Second, while outcome assessors and data analysts will be blinded, the inherent nature of physical interventions such as EECP and ETT training precludes the blinding of participants and therapists, which may introduce potential performance bias. To mitigate this, standardized treatment protocols and objective outcome measures have been strictly implemented. Finally, the 7-week follow-up period focuses primarily on early subacute recovery; thus, the long-term sustainability of the observed functional improvements and their impact on chronic functional status remain to be elucidated in subsequent longitudinal studies.

### Trial status

Patient recruitment is ongoing. This study adheres to protocol version 1.0. Following approval by the ethics committee, the finalized protocol was distributed to the study center managers and archived online to ensure transparency and systematic record-keeping. The first patient was enrolled in July 2024, and the trial is anticipated to be concluded by December 2026.
